# Beyond retirement: the influence of work and resilience on successful aging under stress

**DOI:** 10.1186/s41155-026-00406-0

**Published:** 2026-07-21

**Authors:** Ronaldo Gomes-Souza, Anne Caroline dos Santos Maciel, Erika Priscilla de Freitas Hounsell, Julio Cesar Pinto de Souza, Leandro da Silva-Sauer

**Affiliations:** 1 Faculty of Psychology, Federal University of Amazonas, Manaus, Brazil; 2 Laboratory of Psychology, Work and Mental Health (LAPSIC) of Amazonas, Federal University of Amazonas, Manaus, Brazil; 3 Laboratory of Neuroscience, Neuropsychology, and Assessment (LANNA) of Amazonas, Federal University of Amazonas, Manaus, Brazil

**Keywords:** Psychological stress, Adaptive capacity, Work status, Postretirement, Aging well

## Abstract

Work status in later life has been linked to psychological resilience, social engagement, and overall well-being, all of which may shape how individuals experience and manage stress as they age

**Objective** This study examined the moderating effects of resilience and work status on the relationship between perceived stress and successful aging among older adults.

**Methods** Participants were N = 538 brazilian community-dwelling older adults (≥ 60 years) of both sexes. Measures included a sociodemographic questionnaire, the Brief Resilience Scale, the Perceived Stress Scale (independent variable), and the Successful Aging Scale (dependent variable). Resilience and current work status (employed vs. not employed) were tested as moderators using a cross-sectional, quantitative design. Data were analyzed through descriptive and inferential statistics, followed by a moderated moderation analysis.

**Results** The three-way interaction among perceived stress, resilience, and work status was statistically significant (p < .05). Among non-working older adults, perceived stress was negatively associated with successful aging at all levels of resilience, with this association being attenuated at higher levels of resilience. In contrast, among working older adults, perceived stress was not significantly associated with successful aging at any level of resilience.

**Conclusion** Perceived stress is negatively associated with successful aging, moderated by resilience and work status. Resilience buffered this association among non-working older adults, whereas no significant association was observed among those employed. These findings suggest that employment may alter stress processes and highlight the importance of supporting adaptive transitions into retirement and access to psychosocial resources.

## Introduction

Population aging is a global phenomenon with important social and economic implications. In Brazil, the 2022 Demographic Census reported 31.2 million older adults, representing 14.7% of the population, and life expectancy is projected to reach 81 years by 2060 (IBGE, 2023). Sustained participation in active life, especially in the labor market, is a major societal challenge and highlights the need for strategies that promote both adequate working conditions and successful aging (Pak et al., 2023). For many, continuing to work is a necessity driven by socioeconomic conditions and increased longevity, while also contributing to quality of life and well-being (Costa et al., 2018).

Labor force participation among older adults has increased worldwide. Individuals aged 55 and above accounted for 10.9% of the global workforce in 1995, rising to 16.3% by 2020 (International Labour Organization [ILO], 2024). In South Korea, 24.5% of adults aged 70 and older remained economically active in early 2024 (The Straits Times, 2024). In Brazil, over 13 million people aged 50 or more were employed in 2024 (Brazilian National Review, 2024). This trend raises important questions about how continued work relates to psychosocial factors such as stress and resilience and how these dynamics influence successful aging.

At the same time, the demands of the work environment, combined with natural aging processes, may increase the risk of psychological distress, which is particularly common among older adults who remain active in the workforce (Malik et al., 2022 ; Zábó et al., 2025).

Occupational stress has been shown to negatively affect mental health, well-being, and workplace functioning across different age groups (Ko et al., 2025; Nawata, 2024).

Stress is conceptualized as a particular relationship between the individual and the environment, evaluated by the person as taxing or exceeding their resources and endangering their well-being (Surachman & Almeida, 2018). This definition, originally proposed by Lazarus and Folkman (1984), emphasizes the importance of individual appraisal in the experience of stress. In older adults, this perspective is particularly relevant, as age-specific challenges can influence both the evaluation of stressors and the coping strategies employed.

Aging is often accompanied by events that may act as stressors, including retirement, changes in daily structure, health challenges, and difficulties in psychological adjustment and emotion regulation (Hed et al., 2024; Ferguson et al., 2025). These experiences can compromise successful aging, which is conceptualized as the maintenance of physical and mental health, autonomy, social integration, and economic independence (Rodrigues et al., 2023). Importantly, successful aging extends beyond health outcomes to include the continuity of meaningful social roles, such as work, which provides older adults with a sense of purpose, usefulness, and identity (Alvarenga et al., 2009).

Literature consistently demonstrates that resilience acts as a protective factor against perceived stress, attenuating its negative effects on older adults’ mental health (da Silva-Sauer et al., 2021; Moreno-Agostino et al., 2021; Upasen et al., 2024). Recent evidence further highlights that higher psychological resilience in older adults is associated with reduced mortality risk and improved subjective well-being (Zhang et al., 2024). Previous research indicates that older adults with higher resilience show better emotional adaptation to adversity (Smith et al., 2008), facilitate emotional regulation (Ong et al., 2009), and constitute a key factor supporting successful aging by promoting adaptive functioning and engagement with life (Merchant et al., 2022).

However, various individual and contextual constructs may mediate or moderate the relationship between resilience and positive outcomes. For example, a prior community-based study observed that regular physical activity and higher religiosity levels potentiated resilience effects on mental health, suggesting these factors operate as additional coping and emotional self-regulation resources (da Silva-Sauer et al., 2025). This finding indicates the possibility that other variables may influence how resilience functions in old age, such as current work status, which can represent an important psychosocial resource in the aging context.

Complementarily, engagement in multiple life roles, such as remaining active in the labor market, may generate positive effects across other life domains by expanding the resources available to cope with diverse demands (Greenhaus & Powell, 2006; Hobfoll et al., 2018).

Considering the growing participation of older adults in the labor market, it becomes essential to understand how work influences the aging process. According to the Conservation of Resources Theory (Hobfoll et al., 2018), individuals strive to preserve, protect, and accumulate resources that help them cope with stress and maintain well-being. Within this framework, work can serve as an important source of psychosocial resources, such as structure, purpose, and social support, that facilitate coping with stressful demands in later life. At the same time, resilience has been widely recognized as a protective factor in the face of adversity (Smith et al., 2008; Ong et al., 2009), contributing to more adaptive trajectories of aging (Cosco et al., 2017).

Nonetheless, few studies have examined the combined moderating effects of work status and resilience on the relationship between perceived stress and successful aging, particularly among older populations. Moreover, engagement in social activities such as volunteering has been shown to sustain a sense of social utility, correlating with better psychological health, lower depression risk, and decreased mortality rates (Gil-Lacruz et al., 2019). For instance, research in Germany demonstrated that volunteering among older adults is linked to reduced hospitalizations and increased adherence to preventive healthcare services, suggesting positive effects on both psychological and functional health (Hajek et al., 2019). Meta-analysis further confirms volunteering’s association with longevity and well-being, positioning it as a genuine form of social utility that benefits aging populations (Okun & Kim, 2016).

Beyond voluntary activities, engagement in paid or productive work confers on older adults not only social support and routine structure but also reinforces self-esteem, identity, and purpose, key components linked to psychological resilience. A recent systematic review on labor engagement among older populations underscored that emotional involvement in work contributes to well-being and positive adaptation in later life (Truxillo et al., 2023). Moreover, longitudinal studies show that socially active older adults report higher life satisfaction, reduced depression or isolation, and greater longevity (Anderson et al., 2014). This body of evidence supports the concept of “productive aging,” which posits that activities contributing to society help maintain self-esteem and mental health in older adults (Morrow-Howell, 2010).

Importantly, even low-intensity volunteer engagement (1 to 4 h per week) has been shown to decelerate biological aging and alleviate stress, underscoring the concrete health benefits of maintaining a sense of usefulness in later life (Cheng et al., 2024). Given the growing prevalence of older adults who remain professionally active, investigating how paid work status interacts with resilience to influence successful aging is a timely and necessary endeavor.

This study is grounded in Lazarus and Folkman’s (1984) transactional model of stress, which highlights the role of cognitive appraisal and coping processes in mediating the effects of stressors. Building on this framework, the present research aims to examine the relationship between perceived stress and successful aging, testing resilience and work status as moderators. Specifically, we investigate whether resilience buffers the adverse effects of perceived stress on successful aging and whether this buffering varies according to older adults’ engagement in paid work. We hypothesize that resilience mitigates the negative impact of perceived stress on successful aging and that this protective effect is amplified among those who remain professionally active.

Understanding these interactions is critical given the growing older population and the increasing number of seniors in the workforce, which present new challenges for public health and social policy. Identifying psychosocial factors that promote active and healthy aging can inform targeted interventions to enhance well-being of older adults. Furthermore, this study contributes to addressing research gaps concerning the combined influence of stress, resilience, and occupational status on aging outcomes, particularly within underrepresented Latin American populations.

## Method

### Participants

The final sample consisted of 538 older adults aged between 60 and 101 years (*M* = 69.72, *SD* = 7.93), of whom 41.4% were men and 58.6% were women. Inclusion criteria were: (1) being 60 years of age or older, in accordance with the World Health Organization’s definition of older adults in developing countries; (2) the ability to understand and respond to questions, either in written form (for literate individuals) or orally (for illiterate individuals); and (3) living in the community (i.e., not institutionalized and without a history of dementia).

A priori power estimates using G*Power 3.1 (Faul et al., 2009) indicated that approximately 550 participants would provide sufficient statistical power (1 − β > 0.80) to detect small interaction effects (f² ≈ 0.02) in moderated moderation models, even when controlling for multiple covariates. These thresholds are consistent with Cohen (1988) guidelines for effect sizes in multiple regression.

### Measures

#### Sociodemographic data

Demographic and socioeconomic information was collected, including age, sex (male and, female), educational level (years), marital status (single, married, widowed, divorced or separated and, other), and self-reported race/color (Asian, White, Brown, Black, and Indigenous). Monthly income was categorized in minimum wages (≤ 1; 2–3; 4–5; 6–7; 8–9 and; ≥10). Participants also reported their engagement in volunteer activities (yes/no) and frequency of physical activity (does not engage; 1–2 times/week; 3 times/week and; ≥4 times/week), as well as their perceived physical health. Employment status was assessed by asking participants whether they were currently working and was coded as 1 (working) and 2 (not working). This variable was included as a moderator in the proposed model.

#### Perceived stress

Self-reported stress was assessed using the 10-item Perceived Stress Scale (PSS-10; Cohen & Williamson, 1988). The PSS-10 is a widely used self-report instrument designed to assess perceived stress over the past four weeks, focusing on the extent to which individuals appraise their lives as unpredictable, uncontrollable, and overloaded (“how unpredictable, uncontrollable, and overloaded respondents find their lives during the last month”; Cohen & Williamson, 1988, p. 34). Items are rated on a 5-point Likert scale ranging from 0 (never) to 4 (very often). Four positively worded items (items 4, 5, 7, and 8) are reverse scored, and total scores are obtained by summing all items, ranging from 0 to 40, with higher scores indicating greater perceived stress. The Brazilian Portuguese version of the PSS-10 has demonstrated satisfactory psychometric properties (Reis et al., 2010). In the present study, the scale showed adequate internal consistency (ω = 0.83). Perceived stress was included as the independent variable in the moderation model.

#### Resilience

The Brief Resilience Scale (BRS; Smith et al., 2008) was used to assess individuals’ capacity to recover from stress. The BRS comprises six items, three of which are negatively worded (items 2, 4, and 6; e.g., “It is hard for me to bounce back when something bad happens”). 

Participants indicated their level of agreement with each statement using a 5-point Likert scale ranging from 1 (strongly disagree) to 5 (strongly agree). Higher scores indicate greater resilience. The Brazilian adaptation of the BRS demonstrated adequate psychometric properties in a large sample of older adults (da Silva-Sauer et al. 2020a). Internal consistency in the present study was ω = 0.78. Resilience was conceptualized as one of the moderators in the proposed model.

#### Successful aging

The Successful Aging Scale (SAS; Reker, 2009) was originally developed to assess the subjective dimension of successful aging. The Brazilian Portuguese version (SAS-B; da Silva-Sauer et al., 2020b) retained the same three-factor structure but removed item 11 to improve model fit. The three domains in the SAS-B are Healthy Lifestyle (four items: 01, 07, 08, 13), Adaptive Coping (four items: 02, 03, 12, 14), and Engagement with Life (five items: 04, 05, 06, 09, 10). Items are rated on a 7-point Likert scale ranging from 1 (strongly agree) to 7 (strongly disagree), with higher total scores (ranging from 13 to 91) reflecting more positive perceptions of aging. In the present study, the total SAS-B score was used, with internal consistency McDonald’s ω = 0.81. Successful aging was considered the dependent variable in the proposed model.

#### Psychological distress

The Hospital Anxiety and Depression Scale (HADS; Zigmond & Snaith, 1983) is a 14-item instrument with a 4-point response format designed to assess emotional distress over the past week. The scale evaluates two types of symptoms within a two-factor structure: anxiety and depression. The Brazilian version of the HADS was used in this study (Botega et al., 1995). The HADS total score was included to assess psychological distress and demonstrated adequate reliability in the present sample (α = 0.84). Psychological distress was entered as a covariate to account for general emotional symptoms, allowing the estimation of the unique effect of perceived stress.

#### Social support

The Social Support Questionnaire (SSQ; Sarason et al., 1983) assesses both sources of social support and participants’ satisfaction with the support received. Given variability in specific sources of support across items and participants, this study focused on the satisfaction subscale (SSQ-S), which comprises six items rated on a 6-point Likert scale ranging from 1 (very dissatisfied) to 6 (very satisfied). The Portuguese version of the SSQ was employed (Matsukura et al., 2002). Adequate reliability was observed in our sample (α = 0.88). Social support satisfaction was included as a covariate in the proposed model.

### Procedure

This was an exploratory cross-sectional study designed to examine the relationships among variables at a single point in time, thereby enabling the analysis of moderators within the observed associations. Given the cross-sectional nature of the design, the findings reflect associations rather than causal effects, serving as an initial empirical basis for future longitudinal investigations.

The study was approved by the Research Ethics Committee of the Federal University of Paraíba, via Plataforma Brasil of the Brazilian National Research Ethics Commission (CONEP), under protocol number 341,967. All procedures were conducted in accordance with ethical standards for research involving human participants, and data collection commenced only after ethical approval had been obtained.

Data collection was conducted across multiple institutions, including neighborhood associations, dental clinics, community centers for older adults, educational institutions, occupational therapy clinics, churches, and commercial establishments, located in three Brazilian states: Paraíba (João Pessoa, Cabedelo, and Itapororoca), Pernambuco (Ipojuca and Recife), and Rio Grande do Norte (Ceará-Mirim).

The procedure began with an initial presentation by the research team to the managers and/or supervisors of each site, during which the objectives of the study were explained and information about institutional routines was gathered.

Participants were approached informally and voluntarily agreed to take part after being informed about the aims of the study, the procedures involved, data confidentiality, and anonymity. After providing written informed consent, eligibility was confirmed by verifying participants’ age, and the questionnaires were administered.

The assessment protocol included, in sequence: the sociodemographic questionnaire, the SAS-B, the BRS, the PSS-10, the HADS, and the SSQ-S. The estimated completion time for the full assessment was 15–20 min.

### Data analysis

Descriptive inferential statistics (means, standard deviations, and Pearson’s correlations) were computed for all sociodemographic variables and study instruments. Internal consistency reliability was assessed using McDonald’s omega coefficient (ω) and Cronbach’s alpha (α), depending on the coefficient originally used for each measure.

A moderated moderation analysis was then conducted to examine the relationship between perceived stress (independent variable) and successful aging (dependent variable), with resilience as the first moderator and employment status as the second moderator. The analysis controlled for age, sex, education level, marital status, race/color, social support, physical activity, income, and psychological distress. Assumptions of linearity, normality of residuals, and independence of errors were verified and met.

All analyses were performed in the Statistical Package for the Social Sciences (SPSS) using the PROCESS macro (Model 3), version 4.3 (Hayes, 2018). Following Hayes’ recommendations, the model employed heteroskedasticity-consistent standard errors (HC0), mean-centered continuous variables, and 10,000 bootstrap samples with 95% confidence intervals. The level of statistical significance was set at *p* < .05.

## Results

The sample comprised 538 participants, of whom 41.4% were male and 58.6% were female. Most participants were married (52.8%), followed by widowed (21.9%) and divorced or separated (15.2%). Regarding race/color, 47.2% identified as White and 42.8% as Brown/mixed, with smaller proportions identifying as Black (6.7%), Asian (3.0%), or Indigenous (0.4%). Mean scores for the total sample were as follows: social support (M = 30.3, SD = 4.7), perceived stress (M = 13.4, SD = 6.9), psychological distress (M = 9.9, SD = 6.4), resilience (M = 20.6, SD = 3.8), and successful aging (M = 74.7, SD = 9.5). Table 1 presents the descriptive statistics of the study variables stratified by employment status.

**Table 1:** Descriptive statistics by employment status

Variables	Statistics M (SD) or *n*	Employment Status		Total sample (*N* = 538)
		Employed (*N* = 177)	Not Employed (*N* = 361)	
Age	M (SD)	66.16 (5.7)	71.46(8.2)	69.72(7.9)
Years of education	M (SD)	11.56 (5.1)	8.98 (5.1)	9.83(5.2)
Sex (n)				
Male	n	91	132	223
Female	n	86	229	315
Self-reported race/color				
White	n	92	162	254
Black	n	12	24	36
Brown	n	67	163	230
Indigenous	n	2	0	2
Asian	n	4	12	16
Marital status				
Single	n	15	27	42
Married	n	108	176	284
Widowed	n	20	98	118
Divorced or separated	n	27	55	82
Other	n	7	5	12
Volunteer activity				
Yes	n	68	107	175
No	n	109	254	363
Physical activity				
No physical activity	n	55	143	198
1–2 times per week	n	34	116	150
3 times per week	n	35	40	75
4 or more times per week	n	53	62	115
Income				
≤ 1 minimum wage	n	8	29	37
2–3 minimum wages	n	43	173	216
4–5 minimum wages	n	39	65	104
6–7 minimum wages	n	19	34	53
8–9 minimum wages	n	33	36	69
≥ 10 minimum wages	n	35	22	57
BRS	M (SD)	21.70(3.4)	20.03(3.8)	20.58(3.8)
PSS-10	M (SD)	11.00(6.5)	14.61(6.8)	13.42(6.9)
HADS	M (SD)	7.98 (5.66)	10.89(6.55)	9.93(6.4)
SSQ-S	M (SD)	30.87(4.4)	30.06(4.8)	30.32(4.7)
SAS-B	M (SD)	78.31(7.3)	72.92(10.0)	74.69(9.5)

Continuous variables are presented as *M* (*SD*), and categorical variables as absolute frequencies (*n*)

*N* = total sample size

Pearson correlation analyses were conducted to examine associations among the study variables (Table 2). Psychological distress was positively correlated with perceived stress (PSS-10; *r* = .70, *p* < .001) and negatively correlated with resilience (BRS; *r* = − .48, *p* < .001), social support (SSQ-S; *r* = − .33, *p* < .001), and successful aging (SAS-B; *r* = − .50, *p* < .001). Perceived stress was negatively associated with resilience (*r* = − .53, *p* < .001), social support (*r* = − .30, *p* < .001), and successful aging (*r* = − .52, *p* < .001). Positive correlations were observed between resilience and social support (*r* = .24, *p* < .001), resilience and successful aging (*r* = .41, *p* < .001), and social support and successful aging (*r* = .27, *p* < .001).

**Table 2:** Pearson correlations among instruments

Variable	1	2	3	4
1. HADS	1			
2. BRS	−0.483**	1		
3. PSS-10	0.700**	−0.530**	1	
4. SSQ-S	−0.326**	0.242**	−0.295**	1
5. SAS-B	−0.495**	0.412**	−0.520**	0.270**

Pearson correlation coefficients (r) are presented

*HADS*  Hospital Anxiety and Depression Scale, *BRS*  Brief Resilience Scale, *PSS-10*  Perceived Stress Scale, *SSQ-S*  Social Support Questionnaire, satisfaction subscale, *SAS-B*  Successful Aging Scale

** indicates *p* < .001

### Moderated moderation analysis

**Fig. 1:**
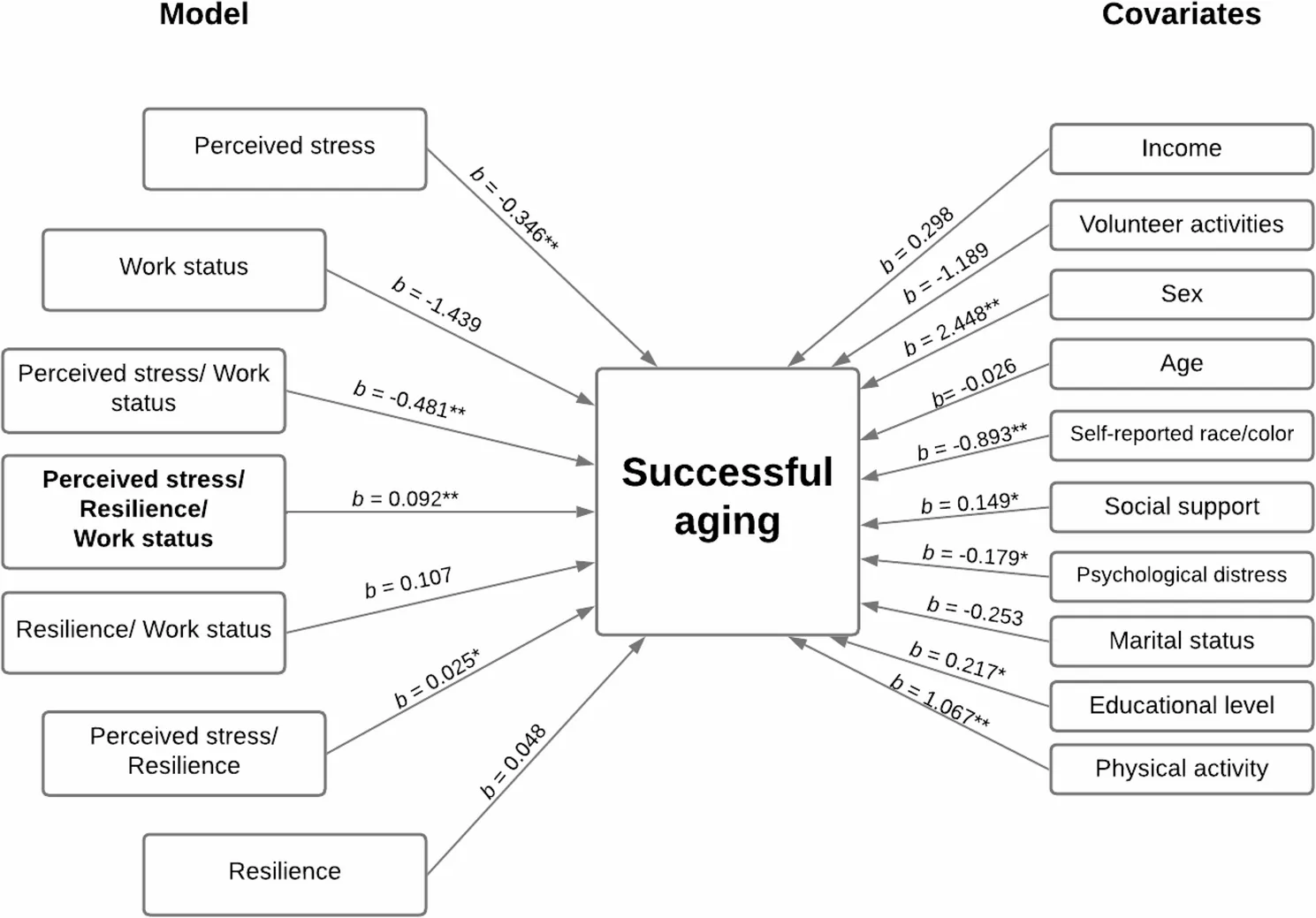
Moderated moderation model predicting successful aging. Note. Path diagram of the moderated moderation model (PROCESS Model 3) with unstandardized regression coefficients (b). Interaction effects significant at p < .05 (*) and p < .001 (**). All sociodemographic and psychosocial covariates (age, sex, educational level, marital status, self-reported race/color, social support, psychological distress, physical activity, income, and participation in volunteer activities) were included in the model as direct predictors of successful aging. Standardized coefficients are not presented, as interpretation of unstandardized coefficients is more appropriate in models including interaction terms

A moderated moderation model was tested to examine whether the association between perceived stress and successful aging was jointly moderated by resilience and employment status, controlling for relevant sociodemographic and psychosocial covariates. The full model is presented in Fig. 1. The overall model was statistically significant, F(17, 514) = 27.17, *p* < .001, explaining 47.3% of the variance in successful aging (*R* = .69, R² = 0.47).

The three-way interaction among perceived stress, resilience, and employment status was statistically significant, ΔR² = 0.011, F(1, 514) = 10.48, *p* < .01. Perceived stress showed a significant negative main effect on successful aging (b = − 0.35, *p* < .001), whereas resilience (b = 0.05, *p* > .05) and employment status (b = − 1.44, *p* > .05) were not significant.

Among the interaction terms, the interaction between perceived stress and resilience was significant (b = 0.03, *p* < .05), as was the interaction between perceived stress and employment status (b = − 0.48, *p* < .001). The interaction between resilience and employment status was not significant (b = 0.11, *p* > .05). The three-way interaction remained significant (b = 0.09, *p* < .01).

**Fig. 2:**
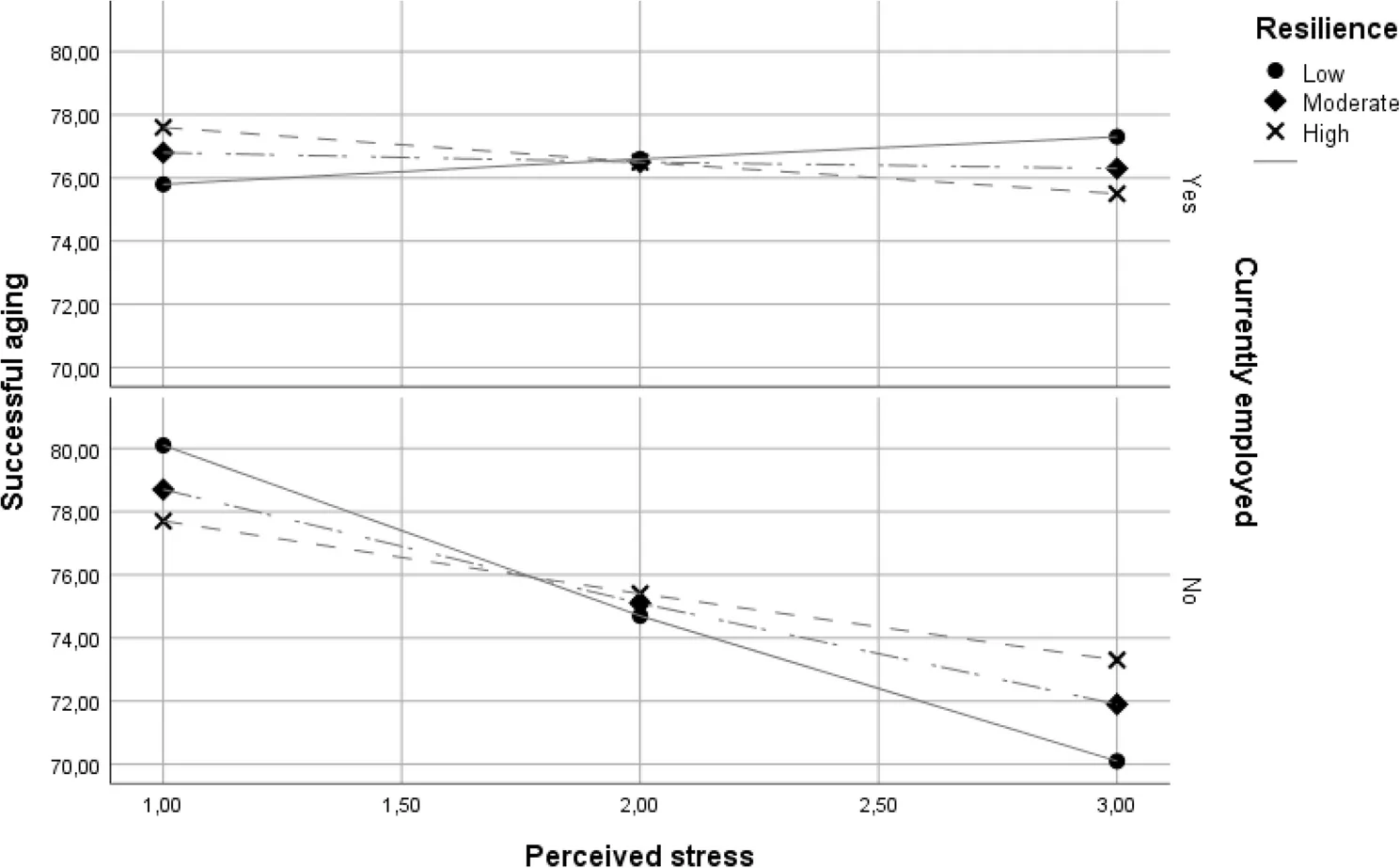
Visual representation of the conditional effects in the model. Note. The y-axis represents scores on the successful aging scale, while the x-axis displays the three levels of perceived stress: 1 = low, 2 = moderate, and 3 = high. The plots are divided by work status: the upper panel corresponds to older adults who are currently employed, and the lower panel refers to those who reported not working

Conditional effects were examined at low, moderate, and high levels of resilience for both employed and non-employed participants (see Table 3), and are illustrated in Fig. 2.

**Table 3:** Conditional effects of perceived stress on successful aging by resilience level and employment status

Resilience Level	Employment Status	Effect (b)	SE	t	*p*	LLCI	ULCI
Low	Employed	0.106	0.148	0.718	0.472	−0.184	0.398
	Not Employed	−0.708	0.092	−7.658	< 0.001	−0.890	−0.526
Moderate	Employed	−0.039	0.095	−0.416	0.677	−0.226	0.147
	Not Employed	−0.485	0.081	−5.987	< 0.001	−0.645	−0.326
High	Employed	−0.149	0.111	−1.340	0.180	−0.368	0.069
	Not Employed	−0.318	0.095	−3.350	< 0.001	−0.505	−0.131

*Effect* Unstandardized regression coefficient, *SE*  Standard error, *t*  t-test statistic, *p*  Significance level, *LLCI*  Lower limit of the 95% confidence interval, *ULCI*  Upper limit of the 95% confidence interval

Among non-employed older adults, perceived stress was significantly and negatively associated with successful aging at all levels of resilience (all ps < 0.01). Although the magnitude of this effect decreased as resilience increased, it remained statistically significant. In contrast, among employed older adults, the association between perceived stress and successful aging was not statistically significant at any level of resilience (all ps > 0.05).

## Discussion

This study investigated the moderating role of psychological resilience on the relationship between perceived stress and successful aging among older adults, considering their employment status. The findings indicate that resilience does not uniformly buffer the stress–successful aging association; instead, its protective role emerged specifically among non-employed older adults.

Contrary to our initial expectation that resilience would exert a stronger protective effect among employed older adults, occupational engagement appeared to reduce the extent to which perceived stress translated into evaluations of successful aging. This pattern suggests that the protective role of resilience is context-dependent and that occupational contexts may provide psychosocial resources that partially regulate stress-related processes in later life.

In the fully adjusted model, covariates were included to account for key biopsychosocial conditions underlying the association between perceived stress, resilience, and successful aging. Psychological distress was negatively associated with successful aging, consistent with the transactional model of stress (Lazarus & Folkman, 1984), in which emotional burden reflects maladaptive stress appraisal and coping processes that compromise adaptive aging outcomes (Oshio, 2024). Physical activity was positively associated with successful aging, operating as a behavioral resource that supports functional capacity and autonomy in later life (Wang et al., 2024). Social support was also positively associated with successful aging, reflecting its role as a core relational resource that provides emotional, instrumental, and informational support, functioning as a primary external resource within the resource conservation framework and buffering stress effects in later life (Hobfoll et al., 2018). Educational level was positively associated with successful aging, reflecting cumulative life-course resources that shape cognitive and socioeconomic conditions for adaptation in later life (Passos et al., 2015; Cosco et al., 2017).

Sex and race/color differences further indicate the influence of structural inequalities in aging trajectories in Brazil (Chor & Lima., 2005; da Silva-Sauer et al., 2020b). In contrast, income, marital status, age, and volunteer activity were not significant, suggesting that distal socioeconomic indicators contributed less to successful aging than proximal psychosocial and behavioral resources within the adjusted model.

The present findings indicate a clear divergence between employment groups regarding the stress–successful aging relationship. Among employed older adults, perceived stress was not significantly associated with successful aging at any level of resilience, suggesting that occupational engagement may reorganize the stress–adaptation process by embedding individuals within a broader contextual system that shapes how stress is appraised and translated into aging outcomes. Evidence indicates that job-related stress and psychological symptoms do not translate into functional outcomes in the same way among working versus retired older adults, suggesting that occupational context modifies the pathways through which stress affects cognitive and adaptive functioning (Ferguson et al., 2025). In this setting, work may reduce the psychological impact of stress through interconnected mechanisms grounded in established theoretical perspectives on the role of employment in later life. These include the provision of role-based identity stability and meaning (Truxillo et al., 2023), the structuring of daily routines and time organization (Ekerdt & Koss., 2016), and the facilitation of social integration and contextual coping resources (Bakker & Demerouti, 2007; Greenhaus & Powell, 2006). Together, these processes shape the appraisal through which stress is interpreted, reducing the extent to which it is cognitively perceived as threatening to overall functioning, consistent with stress appraisal theory (Lazarus & Folkman, 1984).

This may limit its downstream effects on successful aging, as continued engagement in work has been associated with the maintenance of social roles and identity in later life (Alvarenga et al., 2009), higher levels of perceived quality of life and well-being (Costa et al., 2018), and more favorable trajectories of work ability and motivation among older workers (Pak et al., 2023). Together, these findings suggest that occupational engagement contributes to a broader system of psychosocial resources that can buffer or reorganize the impact of stress on aging outcomes, in line with the Work–Family Enrichment Theory (Greenhaus & Powell, 2006) and resource-based perspectives emphasizing contextual regulation of psychological demands (Hobfoll et al., 2018). Rather than indicating an absence of association, this pattern may reflect a reduction in the extent to which stress appraisal directly influences evaluations of overall functioning, possibly because occupational contexts provide external psychosocial resources that support adaptation.

From this perspective, resilience appeared to play a less central role in shaping the relationship between stress and successful aging among employed older adults, possibly because occupational environments provide contextual psychosocial resources that reduce reliance on individual coping capacities. This interpretation aligns with evidence that psychosocial resources operate as integrated systems, consistent with a systems-based view of psychosocial functioning, in which contextual conditions can absorb or redistribute the effects of vulnerability factors (da Silva-Sauer et al., 2026a). One possible interpretation is that occupational environments provide contextual resources that reduce reliance on individual resilience for managing stress-related experiences. In this context, resilience may not differentially condition the association between stress and successful aging among employed older adults because some adaptive functions may be supported by external social and occupational structures.

In contrast, among non-employed older adults, perceived stress was significantly associated with lower levels of successful aging, and this association was moderated by resilience, such that higher resilience attenuated the negative impact of stress. This pattern suggests that, in the absence of structured occupational roles that organize daily meaning, emotional regulation, and social engagement, stress appraisal is more directly translated into evaluations of aging outcomes. In this context, adaptation to aging-related stressors becomes more dependent on individual regulatory capacities, such as emotion regulation and resilience (Hed et al., 2024), and stress may be less buffered by external role-based structures, thereby exerting a more proximal influence on psychological and subjective aging processes.

Consistent with Conservation of Resources Theory, when external resource reservoirs are limited, individuals rely more heavily on internal regulatory systems to sustain adaptation (Hobfoll et al., 2018). Here, resilience functions as a central compensatory mechanism by supporting cognitive reappraisal of stress and maintaining emotional coherence in the absence of stable external anchors. This interpretation converges with evidence that successful aging emerges from dynamic systems of psychological and social resources rather than isolated traits (da Silva-Sauer et al., 2026a), and that internal resources become particularly salient when external sources of meaning, identity, and emotional regulation are reduced (da Silva-Sauer et al., 2026b). In this configuration, resilience operates less as a trait and more as an active regulatory process within a constrained resource environment. Importantly, this differential pattern clarifies the divergence between groups: whereas in employed older adults occupational contexts may regulate stress appraisal before it escalates into subjective aging evaluations, in non-employed individuals resilience becomes the primary mechanism through which stress is cognitively and emotionally processed into aging-related outcomes. In the absence of occupational structure, adaptation to aging-related stressors becomes more dependent on individual regulatory capacities, such as emotion regulation and resilience (Hed et al., 2024).

The findings of this study have important social implications. They highlight the need for public policies and community programs that recognize employment status and psychological resilience as key factors in promoting successful aging. Continued engagement in meaningful work may provide psychosocial resources that support well-being and reduce the impact of stress. For those who are no longer working, interventions should focus on strengthening resilience through social support, purposeful activities, and access to mental health resources.

Importantly, these results do not suggest delaying retirement, but rather emphasize the need to support adaptive transitions by preserving key psychosocial functions of work, such as daily structure, sense of purpose, and social connection. Facilitating these processes may help ensure that retirement is experienced as a period of continued engagement and well-being.

### Limitations and future directions

This study has limitations that should be acknowledged. First, its cross-sectional design limits the ability to establish causal relationships among the variables and raises the possibility of reverse causality or unmeasured third variables influencing the observed associations (Pak et al., 2023). For example, it is plausible that individuals with higher resilience are more likely to remain employed or perceive lower stress levels, rather than employment status directly shaping resilience. Nonetheless, cross-sectional designs are appropriate for exploratory research aimed at identifying potential mechanisms linking social and psychological constructs. Grounded in Lazarus and Folkman’s (1984) transactional model of stress, which emphasizes the role of cognitive appraisal and coping processes in mediating the effects of stressors, this study allowed for the identification of theoretically plausible patterns and associations that can inform future longitudinal investigations. As argued by O’Laughlin et al. (2018), cross-sectional data models, when cautiously interpreted, can serve as valuable starting points for generating hypotheses and justifying more robust longitudinal studies in the future.

Second, the dichotomous operationalization of work status as “working” versus “not working” may obscure some nuances, such as duration of employment, number of working hours, or remuneration level. However, even this simple categorization likely captures the meaningful distinction between being actively engaged in work and not working, which is relevant for understanding the links between stress, resilience, and successful aging. Future research incorporating more detailed occupational indicators could provide a more nuanced understanding of how specific work characteristics shape well-being in later life.

Additionally, although physical activity was included as a covariate and was significantly associated with successful aging, it does not fully capture baseline physical health. Thus, the potential influence of the healthy worker effect cannot be ruled out. Finally, although sociodemographic covariates were statistically controlled, other potentially relevant factors, including physical health conditions, job satisfaction, and the quality of social relationships, were not assessed and may contribute to the observed effects. Including these variables in future models could help minimize omitted-variable bias and strengthen theoretical accuracy.

Despite these limitations, the study offers novel evidence regarding the interactive roles of work status and psychological resilience in successful aging. The findings provide an empirically supported and conceptually coherent framework, grounded in a well-established theoretical model, for understanding how older adults mobilize internal and external resources to manage stress. Future research using longitudinal approaches, more detailed occupational measures, and diverse samples can further elucidate these complex dynamics and guide the development of interventions and public policies that foster resilience and well-being in older adults across social and occupational settings.

## Conclusion

The findings indicate that psychological resilience and employment status jointly shape the association between perceived stress and successful aging, revealing that the protective role of resilience is context-dependent. Among non-employed older adults, resilience functioned as an important adaptive resource, attenuating the negative impact of stress, whereas among employed individuals this association was not observed. This pattern suggests that occupational contexts may influence how stress is appraised and translated into aging outcomes by providing stable psychosocial resources that support adaptation in later life. In the absence of such structures, resilience may become more central for maintaining adaptation under stressful conditions. Overall, the findings highlight that successful aging is sustained through dynamic interactions between internal psychological capacities and external contextual resources. The results underscore the importance of supporting adaptive transitions to retirement by preserving opportunities for social participation, meaningful engagement, and access to psychosocial resources in later life.

## Data Availability

The data that support the findings of this study are not publicly available due to privacy and confidentiality restrictions but are available from the corresponding author upon reasonable request.
